# Uniparental mitochondrial DNA inheritance is not affected in *Ustilago maydis Δatg11* mutants blocked in mitophagy

**DOI:** 10.1186/s12866-015-0358-z

**Published:** 2015-02-06

**Authors:** Gaby Wagner-Vogel, Frauke Lämmer, Jörg Kämper, Christoph W Basse

**Affiliations:** Department of Genetics, Institute for Applied Biosciences of the Karlsruhe Institute of Technology (KIT), Karlsruhe, Germany

**Keywords:** Mitophagy, Uniparental mitochondrial inheritance, mtDNA, *Ustilago maydis*, Fungi, Atg11

## Abstract

**Background:**

Maternal or uniparental inheritance (UPI) of mitochondria is generally observed in sexual eukaryotes, however, the underlying mechanisms are diverse and largely unknown. Recently, based on the use of mutants blocked in autophagy, it has been demonstrated that autophagy is required for strict maternal inheritance in the nematode *Caenorhabditis elegans.* Uniparental mitochondrial DNA (mtDNA) inheritance has been well documented for numerous fungal species, and in particular, has been shown to be genetically governed by the mating-type loci in the isogamous species *Cryptococcus neoformans*, *Phycomyces blakesleeanus* and *Ustilago maydis*. Previously, we have shown that the *a2* mating-type locus gene *lga2* is decisive for UPI during sexual development of *U. maydis*. In axenic culture, conditional overexpression of *lga2* triggers efficient loss of mtDNA as well as mitophagy. To assess a functional relationship, we have investigated UPI in *U. maydis Δatg11* mutants, which are blocked in mitophagy.

**Results:**

This study has revealed that *Δatg11* mutants are not affected in pathogenic development and this has allowed us to analyse UPI under comparable developmental conditions between mating-compatible wild-type and mutant strain combinations. Explicitly, we have examined two independent strain combinations that gave rise to different efficiencies of UPI. We demonstrate that in both cases UPI is *atg11*-independent, providing evidence that mitophagy is not critical for UPI in *U. maydis*, even under conditions of strict UPI.

**Conclusions:**

Until now, analysis of a role of mitophagy in UPI has not been reported for microbial species. Our study suggests that selective autophagy does not contribute to UPI in *U. maydis*, but is rather a consequence of selective mtDNA elimination in response to mitochondrial damage.

**Electronic supplementary material:**

The online version of this article (doi:10.1186/s12866-015-0358-z) contains supplementary material, which is available to authorized users.

## Background

Uniparental inheritance (UPI), also known as maternal inheritance in oogamous organisms, is a phenomenon generally observed in sexual eukaryotes [1,2]. UPI of mitochondria means that one parental mitochondrial (mt) DNA population is not transmitted to the sexual progeny. Consequently, this process eliminates heteroplasmy defined as the state in which a mixture of parental mt genomes coexists in a zygote. Elimination can occur either prior to fertilization (or mating in the case of isogameous species), during or after fertilization [3]. The underlying mechanisms, however, are diverse and little understood [4-6]. Recently, two studies have provided molecular evidence that autophagy is involved in selective elimination of sperm-associated mtDNA in fertilized eggs of the nematode *Caenorhabditis elegans* [7-10]. Autophagy is a cellular degradation process by which cell constituents including organelles are enclosed in double-membrane vesicles termed autophagosomes for subsequent disposal in lysosomes or vacuoles [11]. Hereby, the ubiquitin-like protein Atg8 (termed LC3 in mammals or LGG-1/2 in *C. elegans*) is required for autophagosome formation and essential for non-selective (bulk) as well as selective autophagy such as autophagy of mitochondria (mitophagy) [12]. In one study, both *lgg-1* and *lgg-2* were silenced in hermaphrodites of *C. elegans*. Interestingly, subsequent fertilization resulted in survival of male-specific mtDNA in the developing embryo [7]. In the other study, hermaphrodites of either *C. elegans* wild-type or *lgg-1* null mutant were fertilized. Again, only in the mutant background paternal inheritance was detected [8]. In both studies, male partners carried a polymorphic mtDNA to specifically detect paternal inheritance by PCR. These studies have pointed out a requirement of autophagy in the effective elimination of paternal mitochondria. Based on the use of *lgg1/2* mutants, however, this study did not address the possibility of whether the observed phenotype specifically relied on the loss of mitophagy.

Previously, we have investigated UPI in the basidiomycete smut fungus *Ustilago maydis* [13]. This fungus can either grow yeast-like by budding or undergo a sexual cycle, which is initiated by fusion of two mating-compatible isogamous cells. Sexual identity is governed by a tetrapolar mating-type system, constituted by the *a* and *b* loci. The *a* locus exists in two alleles, *a1* and *a2*, which encode a pheromone/receptor-based system providing for nonself recognition and fusion of mating partners. The multiallelic *b* locus controls subsequent sexual development of the dikaryon. In addition, completion of the sexual cycle and formation of diploid teliospores is intimately coupled to biotrophic growth within the host plant maize [14,15]. UPI of mtDNA has been detected as post-fusion event during development of dikaryotic cells. As a result, inheritance of *a2*-associated mtDNA (termed m2 mitotype) dominates, while *a1*-associated mtDNA (termed m1 mitotype) is efficiently lost from the progeny. The outcome of UPI is determined by the *a2* mating-type locus genes *lga2* and *rga2*. Specifically, *lga2* controls selective loss of the m1 mitotype, while *rga2* is required for maintenance of the m2 mitotype. Hence, either absence of *lga2* or expression of *rga2* in both mating partners confers biparental inheritance along with formation of recombinant mitotypes [13]. Both *lga2* and *rga2* encode small basic proteins associated with mitochondria, however, their mode of action is elusive [16,17]. Expression of *lga2* is strongly upregulated in dikaryotic cells and remains highly expressed during biotrophic growth [16,18]. Furthermore, conditional overepression of *lga2* triggers mt fragmentation, selective loss of mt nucleic acids as well as mitophagy [16,19]. Together, this suggested that in *U. maydis* UPI resulted from selective degradation of mitochondria, and hence raised the question of whether mitophagy played an underlying role.

Recently, we have demonstrated that *U. maydis Δatg11* mutants are efficiently blocked in mitophagy in response to starvation as well as *lga2*-triggered mt damage. Specifically, *Δatg11* mutants maintained extensive mt fragmentation after *lga2* overexpression, whereas accumulation of mitochondria-targeted eGFP within vacuoles was abolished [19]. Atg11, initially characterized in yeast, is an adaptor protein specifically required for selective autophagy including mitophagy, but is dispensable for bulk autophagy [12,20,21]. For the present investigation, we analysed UPI in dependence of *atg11* in two strain combinations differing in their efficiencies of m1 mitotype elimination. Our analysis provides evidence that mitophagy is not critical for UPI in *U. maydis*.

## Results

### Pathogenic development of mating-compatible *U. maydis Δatg11* mutants

**Figure 1:**
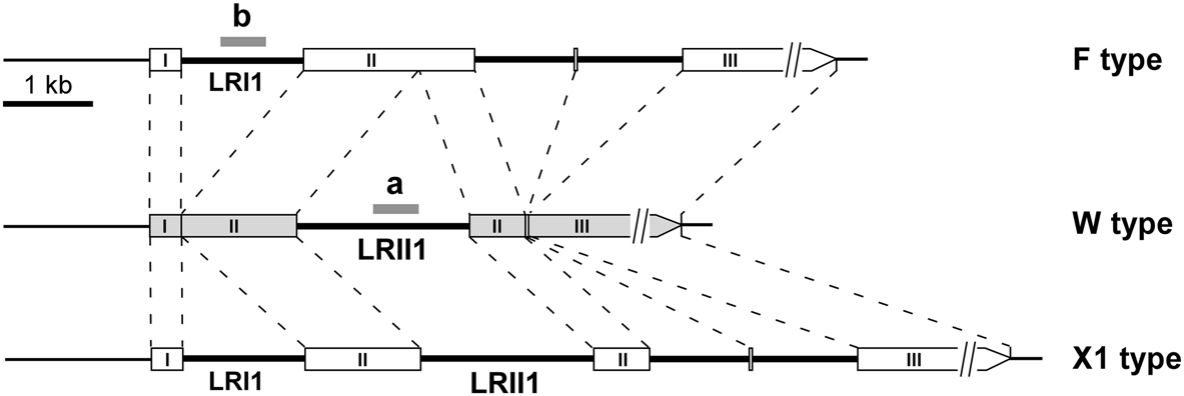
**Physical maps of the F, W and X1 mitotypes.** The schematic shows the arrangement of introns (thick lines) within the polymorphic region of the *U. maydis* LSU rRNA gene (drawn to scale). The three major exon sequences are boxed and numbered from I to III. Stippled lines connect homologous exon sequences. Intron regions amplified by the primer combinations ‘a’ (W type specific) and ‘b’ (F type specific) are marked in grey. The schematic is adapted from [13] with permission granted by the Genetics Society of America.

In a previous study we have shown that *U. maydis Δatg11* null mutants are blocked in mitophagy [19]. To assess a possible influence of mitophagy on UPI, we generated mating-compatible *Δatg11* null mutant strains differing in their mitotypes (Additional file 1: Table S1). The presence of *mtGFP* or *mtRFP* reporter constructs, allowing the detection of mt matrix-targeted green and red fluorescent protein, respectively, was irrelevant for this study, but previously served to assure the absence of mitophagy in FB1 (*a1*) and FB2 (*a2*) *Δatg11* mutant strains under starvation conditions [19]. Physical maps of the parental F and W types and the recombinant X1 type are depicted in Figure 1. The generated *Δatg11* null mutant strains were verified by RFLP (Additional file 2: Figure S1) and PCR analyses as described [19,22] (see Methods). The *U. maydis* strains MF34 and GF5 both carry the W type, but differ in the *a* mating-type. Since our previous investigation has shown that the m2 mitotype dominates over the m1 mitotype this provided for analysis of a possible influence of *atg11* on UPI in reciprocal combinations, in which either inheritance of the F type (combination I: MF34 [*a1*,W] x FB2 [*a2*,F]) or the W type (combination II: FB1 [*a1*,F] x GF5 [*a2*,W]) dominated. Previously, combination I was shown to produce biparental inheritance to a minor extent (<10%) as judged from inheritance of the m1 mitotype as well as the recombinant X1 type to the sexual spore progeny, while mtDNA inheritance in combination II was apparently strictly uniparental based on RFLP analysis [13]. For analysis of UPI, we infected maize plants with combinations I and II in the presence or absence of *atg11*. This study revealed that *Δatg11* mutants were neither affected in pathogenesis (Additional file 3: Figure S2) nor in their ability to produce teliospores (56-63% spore-producing tumours for the wild-type [wt] and *Δatg11* combination I, respectively; n = 18-19). The absence of significant differences in pathogenesis was corroborated by statistical analysis applying a non-parametric Mann–Whitney test (see Methods). This revealed p-values of 0.85 and 1.00 for the (1)/(2) and (3)/(4) data sets, respectively, clearly exceeding the significance level of 0.05 (Additional file 3: Figure S2). Hence, this allowed us to investigate a possible role of mitophagy in UPI under comparable developmental conditions between wt and *Δatg11* mutant strains.

### Analysis of UPI in strain combination I

**Figure 2:**
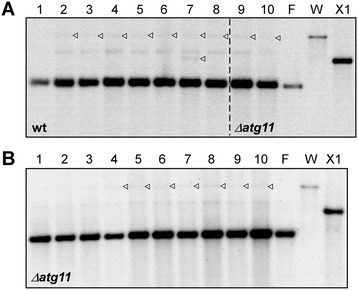
**RFLP analysis to detect mitotypes in wt and** ***Δatg11*** **spores (combination I). A**. HindIII-digested DNA from individual spores from tumour sample wt#5 (MF34/pKS2 × FB2/pMB2-2) (lanes 1–8) and Δatg11#7 (MF34Δatg11/pKS2 × FB2Δatg11/pMB2-2) (lanes 9–10). **B**. Digested DNA from individual spores from tumour sample Δatg11#11 (MF34Δatg11/pKS2 × FB2Δatg11/pMB2-2) (lanes 1–10). **A**,**B**. Triangles mark minor bands indicative for the W and X1 type, respectively. The outer three lanes represent DNA from the corresponding parental strains (F and W type) and from strain BUB7 (X1 type). The 554-bp mt-specific probe directed against the left border of exon II of the LSU rRNA gene (see Figure 1) was used as described [13,16]. Representative images are shown.

For analysis of UPI we firstly focused on strain combination I. For this purpose, total DNA was extracted from germinated teliospores of mature tumour tissue for subsequent RFLP as well as PCR analysis. Explicitly, we analysed the sexual progeny (≥8 spores per single tumour) from several tumours whereby each tumour originated from a different plant either infected with the wt or *Δatg11* mutant strain combination. RFLP analysis revealed predominant inheritance of the m2 mitotype F as well as detection of the X1 type in a minor portion of samples as expected (Figure 2). A quantitative analysis of all signals obtained from RFLP analyses is depicted in Table 1. Pronounced inheritance of the W type was detected only in two tumour samples resulting from the *Δatg11* combination (category a in Table 1). Importantly, this analysis showed comparable RFLP patterns and similar frequencies of m2 mitotype inheritance in the analysed spore progeny irrespective of the presence of *atg11*. Statistical Mann–Whitney test analysis between the two data sets for the wt and *Δatg11* mutant strains revealed a p-value of 0.375 for the F type dominance (column a in Table 1). In addition, the p-values for dominant (column a) and minor (columns b-d) inheritance of the W and X1 types, which are indicative of biparental inheritance, were 0.524 and 0.506, respectively. In conclusion, this rules out that the two data sets are significantly different.

**Table 1:** **Mitotype determination by RFLP analysis (combination I)**

**Tumor sample** ^**1**^	**Spores analysed**	**F type** ^**3**^ **(m2)**				**W type** ^**3**^ **(m1)**				**X1 type** ^**3**^				**F type dominance** ^**4**^
		**a**	**b**	**c**	**d**	**a**	**b**	**c**	**d**	**a**	**b**	**c**	**d**	
wt#1,#2^2^	8	8	0	0	0	0	0	0	1	0	0	0	0	100
wt#4	10	10	0	0	0	0	0	0	0	0	0	0	0	100
wt#5	9	9	0	0	0	0	0	0	4	0	0	0	1	100
wt#6	10	10	0	0	0	0	0	0	0	0	0	0	0	100
wt#7	10	3	0	0	0	0	0	0	6	7	0	0	0	30
wt#8	10	0	2	0	0	0	0	0	2	8	0	2	0	0
wt#9,#10^2^	9	9	0	0	0	0	0	0	0	0	0	0	0	100
**Σ**	66	**49**	**2**	**0**	**0**	**0**	**0**	**0**	**13**	**15**	**0**	**2**	**1**	**74.2**
Δatg11#1	9	8	0	0	0	0	0	0	1	1	0	0	0	88.9
Δatg11#2	10	10	0	0	0	0	0	0	4	0	0	0	0	100
Δatg11#4	8	7	0	1	0	0	0	0	0	0	1	0	1	87.5
Δatg11#7	10	7	0	0	0	3	0	0	0	0	0	0	0	70
Δatg11#8	10	5	0	0	0	4	0	0	0	1	0	0	0	50
Δatg11#11	10	10	0	0	0	0	0	0	1	0	0	0	0	100
Δatg11#12	10	0	0	0	0	0	0	0	0	10	0	0	0	0
**Σ**	67	**47**	**0**	**1**	**0**	**7**	**0**	**0**	**6**	**12**	**1**	**0**	**1**	**70.1**

^1^Wt strain combination: MF34/pKS2 x FB2/pMB2-2; *Δatg11* strain combination: MF34Δatg11/pKS2 x FB2Δatg11/pMB2-2.

^2^The values from these two samples were grouped.

^3^Relative percentages of band intensities are indicated as: a) >97%, b) >50-97%, c) 3-50%, d) <3%.

^4^Values (F type category a) expressed in percentages (%).

### Analysis of UPI in strain combination II

**Figure 3:**
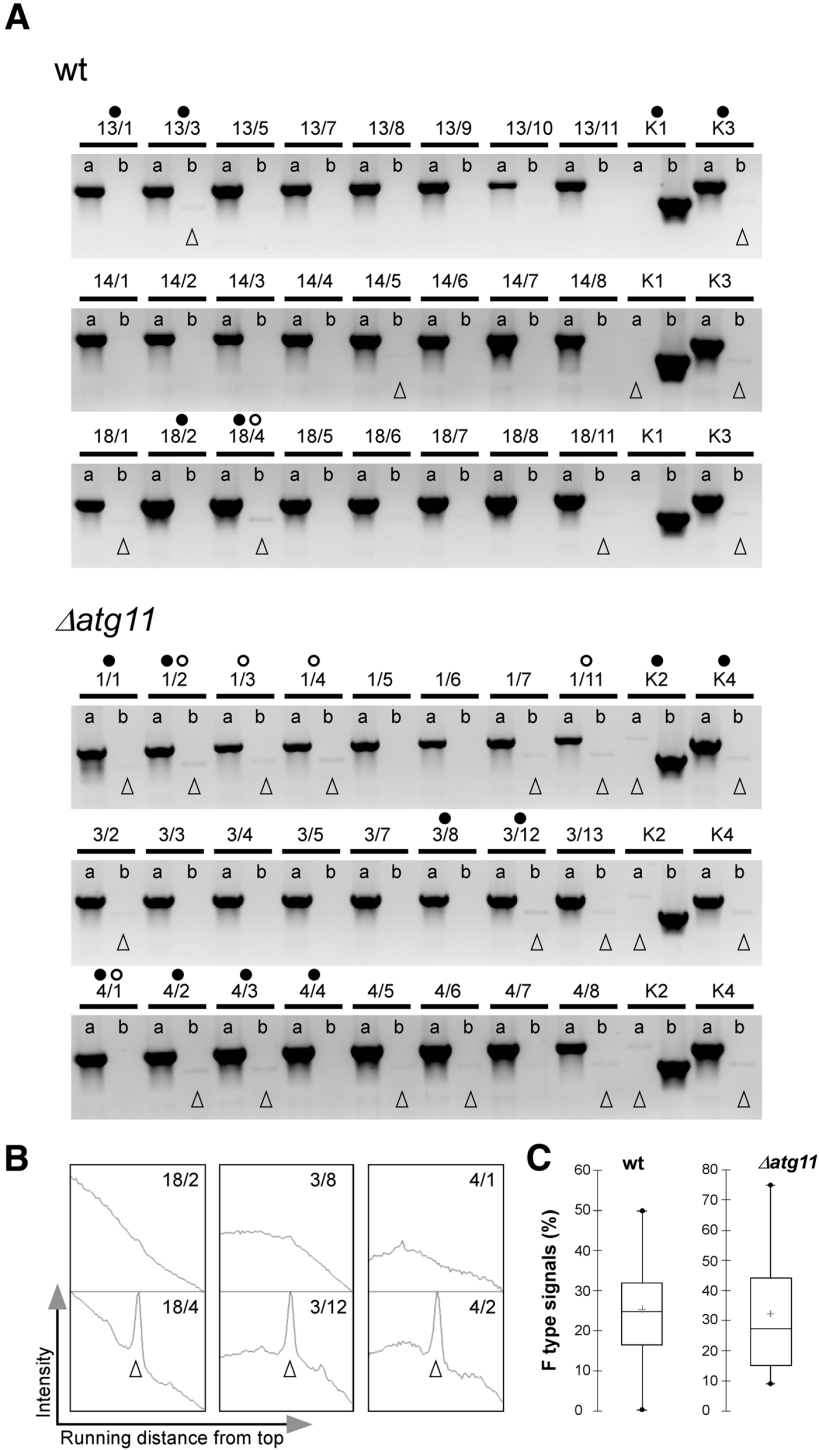
**PCR analysis to detect mitotypes in wt and** ***Δatg11*** **spores (combination II). A**. PCR with DNA from individual spores from each three tumour samples of the wt (FB1/pMB2-2 x GF5/pKS1) and the *Δatg11* mutant strain combination (FB1Δatg11/pMB2-2 x GF5Δatg11/pKS1). Labels ‘a’ and ‘b’ above the lanes refer to the primer combinations to detect the W and F type intron, respectively (see Figure 1). DNA from the parental strains K1 (FB1/pMB2-2), K3 (GF5/pKS1), K2 (FB1Δatg11/pMB2-2), and K4 (GF5Δatg11/pKS1) were included as controls. Open triangles mark faint bands (hardly detectable for probe 1/1) of the non-dominating F type (or W type in case of K1, K2; see Methods). DNA samples that were included in the RFLP analysis are marked by open circles (see Additional file 4: Figure S3). DNA samples that were additionally used for qPCR are marked by filled circles (see Additional file 5: Figure S4). Representative images are shown. **B**. Examples of peak profiles generated by ImageJ of faint F type signals shown in **(A)** and their absence in lanes lacking a corresponding signal. **C**. Boxplot of the data (expressed in percentages) shown in Table 2 (F type column d). Shown are the min./max. range (dots), median (horizontal line in the box), and the mean value (cross). The box contains ca. 50% of all data values ranging in both directions from the median. Note the increased variance (width of the box) for the *Δatg11* mutant.

To assess a possible influence of mitophagy on strict UPI the same experimental approach was applied to strain combination II. To efficiently examine a large number of samples we developed a PCR test based on detection of the mitotype-specific introns LRI1 (F type) and LRII1 (W type) (see Figure 1). Specifically, we used intron-specific primers to avoid competing PCRs in case of heteroplasmy. This approach enabled us to discriminate between UPI (detection of either parental W or F type) from biparental inheritance (detection of both types). For both the mutant and wt strain combination, we analysed 122–123 spores collected from 11–12 tumours each. Mitotype analysis revealed exclusively strict m2 mitotype inheritance in all samples analysed irrespective of the presence of *atg11* (Figure 3A). Only faint signals of the m1 mitotype F were additionally detected in samples of wt and mutant strains (Table 2). As these signals were also associated with the corresponding parental strains used for plant infection these results imply their partial elimination in the spore progeny. Hence, apparently pure homoplasmy was achieved in individual spores irrespective of the presence of *atg11* (Figure 3A-B). Comparison of the data sets referring to leaky F type inheritance (column d in Table 2) between wt and mutant strains by Mann–Whitney test analysis revealed a p-value of 0.576, thus ruling out significant differences. Consistently, boxplot analysis revealed a similar median (central data value) between the *Δatg11* mutant and the wt strain (27.3% vs. 24.7%), but displayed an increased variance (469.7 vs. 236.2) of the data from the *Δatg11* mutant (Figure 3C) in favour of slightly enhanced biparental inheritance (see Discussion).

**Table 2:** **Mitotype determination by PCR analysis (combination II)**

**Tumour sample** ^**1**^	**Spores analysed**	**LRI1 (F type)** ^**2**^ **(m1)**				**LRII1 (W type)** ^**2**^ **(m2)**			
		**a**	**b**	**c**	**d**	**a**	**b**	**c**	**d**
Δatg11#1	8	0	0	0	6	8	0	0	0
Δatg11#2	10	0	0	0	5	10	0	0	0
Δatg11#3	21	0	0	0	8	21	0	0	0
Δatg11#4	10	0	0	0	6	10	0	0	0
Δatg11#5	9	0	0	0	1	9	0	0	0
Δatg11#6	11	0	0	0	3	11	0	0	0
Δatg11#7	11	0	0	0	3	11	0	0	0
Δatg11#8	11	0	0	0	2	11	0	0	0
Δatg11#9	11	0	0	0	1	11	0	0	0
Δatg11#10	10	0	0	0	1	10	0	0	0
Δatg11#11	11	0	0	0	3	11	0	0	0
**Σ**	**123**	**0**	**0**	**0**	**39 (31.7)** ^**3**^	**123**	**0**	**0**	**0**
wt #12	10	0	0	0	5	10	0	0	0
wt #13	10	0	0	0	2	10	0	0	0
wt #14	10	0	0	0	1	10	0	0	0
wt #15	9	0	0	0	2	9	0	0	0
wt #16	8	0	0	0	0	8	0	0	0
wt #17	11	0	0	0	3	11	0	0	0
wt #18	11	0	0	0	5	11	0	0	0
wt #19	11	0	0	0	3	11	0	0	0
wt #20	11	0	0	0	3	11	0	0	0
wt #21	11	0	0	0	5	11	0	0	0
wt #22	9	0	0	0	1	9	0	0	0
wt #23	11	0	0	0	2	11	0	0	0
**Σ**	**122**	**0**	**0**	**0**	**32 (26.2)** ^**3**^	**122**	**0**	**0**	**0**

^1^Wt strain combination: FB1/pMB2-2 x GF5/pKS1; *Δatg11* strain combination: FB1Δatg11/pMB2-2 × GF5Δatg11/pKS1.

^2^Relative percentages of band intensities are indicated as: a) >97%, b) >50-97%, c) 3-50%, d) <3%.

^3^Values expressed in percentages (%).

Next, we performed RFLP analysis to corroborate the finding of *atg11*-independent strict UPI, and furthermore compare the suitability of the selected methods towards detection of leaky biparental inheritance. We therefore included samples showing a faint F type signal as detected by PCR analysis (*e.g.* samples 1/2, 1/3, 1/4; see Figure 3A). However, RFLP analysis revealed exclusive inheritance of the W type, but did not allow detection of faint F type signals (Additional file 4: Figure S3). Hence, this rates the PCR approach more sensitive than the RFLP approach for detecting residual m1 mitotype signals. Consistent with strict UPI, RFLP analysis confirmed the absence of the recombinant X1 type in the sexual progeny (Additional file 4: Figure S3).

### Quantitative determination of the m2/m1 ratios in the sexual progeny

To explicitly determine the efficiency of UPI in strain combination II, we performed a quantitative real-time PCR (qPCR) analysis using DNA from the spore progeny as well as from the parental m2 strains. Furthermore, to rate the suitability of qPCR in terms of its sensitivity to detect leaky biparental inheritance, we used DNA samples from spores that based on PCR analysis had revealed both the presence and absence of faint F type signals (see Figure 3). Primers were directed against the LRII1 or LRI1 introns to determine the ratios of major m2 to minor m1 mitotype. Firstly, this emphasized the strong dominance of W type inheritance in the spore progeny (Additional file 5: Figure S4). In all samples analysed, the calculated [W]/[F] ratios were >10^5^. Secondly, lowest ratios of [W]/[F] were associated with the parental strains as judged from analysing two independent DNA preparations (Additional file 5: Figure S4). As expected, [F]/[W] ratios were similarly pronounced for the parental control strains that displayed F type dominance (Additional file 5: Figure S4A), consistent with comparable efficiencies of the two primer combinations used. Thirdly, occurrence of faint F type bands detected by PCR analysis was not reflected by qPCR analysis; *i.e.*, [W]/[F] ratios of samples from an individual tumour, except for 4/2, were not significantly reduced despite the presence of a faint F type signal (Additional file 5: Figure S4; see Figure 3). This latter finding was surprising based on marked peak profiles calculated from image lanes displaying a faint F type signal (Figure 3B). Hence, this suggests that gel documentation of PCR products is most appropriate for detection of leaky biparental inheritance.

## Discussion

Understanding the mechanisms of UPI is of general importance since these might also account for the elimination of heteroplasmy as caused by accumulation of deleterious mtDNA molecules [6]. In this study we have investigated a possible influence of mitophagy on UPI in the smut fungus *U. maydis*. Recent investigation has indicated a role of autophagy in maternal inheritance in *C. elegans* [7-10]. However, these studies did not address whether elimination of paternal mtDNA specifically relied on mitophagy since LGG-1/2 are generally required for autophagy, and furthermore, are essential for larval development [9]. Based on ubiquitination as well as co-localization of sperm mitochondria with P62 (an autophagic adaptor) and the Atg8 homolog LC3, it was also speculated that autophagy is involved in maternal inheritance in mammals [7,9,10,23]. However, recent evidence does not support such a role of autophagy in mice. It rather appears that maternal inheritance of mtDNA is mainly determined by elimination of sperm mtDNA before fertilization as well as by uneven distribution of sperm mitochondria in embryos [24,25].

To our knowledge, a role of mitophagy in UPI has not yet been addressed in a microbial species. For the current investigation we used *U. maydis Δatg11* mutants, which are blocked in mitophagy under starvation conditions as well as in response to conditional overexpression of *lga2*, a situation that efficiently triggers mitophagy in *U. maydis* [19]. Interestingly, this study has shown that *Δatg11* mutants are not compromised in pathogenic development. Hence, this contrasts from *U. maydis Δatg8* mutants, which are severely attenuated in pathogenic development and hardly incite host tumours [26] (F. Nieto-Jacobo and C. Basse, unpublished). Consequently, *U. maydis Δatg8* mutants did not provide for sexual spores required for analysis of UPI. This finding suggests that bulk autophagy, but not selective autophagy is critical for *U. maydis* pathogenesis. Importantly, the use of *Δatg11* mutants allowed us to examine an influence of mitophagy on UPI under comparable developmental conditions between mutant and wt strains, thus avoiding possible secondary effects.

For this study, we used two strain combinations that yielded UPI with different efficiencies. In particular, combination I, including the W type strain MF34, is somewhat prone to leaky m1 inheritance, thus giving rise to recombinant mitotypes in the sexual offspring, while this was not observed for the W type strain GF5 (combination II) [13]. Currently, the genetic determinants interfering with the efficiency of UPI in *U. maydis* are elusive.

Among the methods used for analysis of UPI, gel documentation of PCR products has proven most consistent in the detection of faint amounts of the m1 mitotype. For combination I, pronounced m2 mitotype dominance along with minor occurrence of the recombinant X1 mitotype was very similar between the spore progeny of the wt and *Δatg11* mutant strains (see Table 1). The exceptional marked inheritance (category a) of the W type in individual tumour samples, as detected for the *Δatg11* combination, has previously also been detected for the respective wt combination, although it rarely occurred (1 of 56 tumours; [13]). For combination II, strict UPI of the m2 mitotype was detected in all samples analysed. In particular, incidences of faint detection of the m1-associated intron were detected to similar extent between wt and mutant strain combinations, although slightly increased leakage of the m1 mitotype in the *Δatg11* strain combination cannot entirely be excluded from the current data (see Table 2). Taken together, these data revealed that, irrespective of the efficiency of UPI, selective elimination of the m1 mitotype was largely unaffected in *Δatg11* mutants. To explicitly quantify the efficiency of UPI in the *U. maydis* strain combination II, ratios of m2/m1 mitotypes in the sexual offspring were determined by qPCR. The values determined for the m2/m1 ratio were in the range of 10^7^-10^8^ for the majority of the analysed spore progeny (see Additional file 5: Figure S4; see Methods) pointing out that the m1 mitotype was grossly eliminated in the sexual progeny. Importantly, the same high ratios were detected for both wt and *Δatg11* mutant strain combinations, further supporting that *atg11* does not affect the efficiency of UPI in *U. maydis*.

Previously, we have shown that Atg11 is required for *lga2*-triggered mitophagy [19]. The current finding of *atg11*-independent UPI implies that *lga2*-induced UPI and mitophagy are separate events. This conclusion is fully consistent with our previous observation that selective loss of mtDNA precedes the onset of mitophagy in response to *lga2* overexpression, suggesting that mitophagy is not the cause, but a secondary response of mtDNA loss aimed to dispose of damaged mitochondria [19]. Nonetheless, a role of mitophagy in UPI of *U. maydis* cannot entirely be excluded. Mitophagy could be involved in disposal of disintegrated mitochondria having maintained residual amounts of DNA as well as their capacity of mt fusion [27]. In addition, it remains to be shown to what extent *atg11*-independent mitophagy operates by default during host infection. Currently, there is no precedence for *atg11*-independent mitophagy reported in yeast [28,29]. For a number of systems, additional proteins mediating mitophagy have been identifed [12,21]. In yeast, this includes the Atg32p protein, a mt outer membrane protein that functions as cargo receptor interacting with Atg11p to mark mitochondria for mitophagy. Furthermore, it has recently been shown that the mt fission factor Dnm1p forms a complex with Atg11p to promote mitophagy in yeast [28]. In light of the finding that in *U. maydis*, *lga2*-triggered mitophagy critically depends on Dnm1 [19], the possibility that Dnm1 contributes to residual mitophagy in *Δatg11* mutants requires further investigation. Apart from selective mitophagy, non-selective macroautophagy or microautophagy-like mitochondria uptake are potential alternative mechanisms of mitochondria disposal [12,21]. Hence, it will be rewarding to investigate mitophagy in the context of UPI in *U. maydis Δatg11* mutants to sort out the fate of disintegrated mitochondria.

Evidence for post-fusion control of parental mtDNA elimination has been provided for numerous microbial species and in a few cases, including the fungal species *Microbotryum violaceum*, *Cryptococcus neoformans, Ustilago maydis* and *Phycomyces blakesleeanus*, underlying control by the mating-type loci has been demonstrated [2-5,30,31]. Confronted with a potential diversity of mechanisms underlying UPI it might be worthwhile to widen the current investigation to further assess a role of mitophagy in establishing homoplasmy.

## Conclusions

Collectively, this study has shown that in *U. maydis* UPI of mtDNA does not require *atg11*-dependent mitophagy. The present work may be particularly useful in providing a framework for understanding mechanistic differences in the regulation of strict UPI between isogamous and oogamous species.

## Methods

### Strains, growth conditions and chemicals

All *U. maydis* strains used in this study are listed in (Additional file 1: Table S1). *U. maydis Δatg11* mutants were generated by PCR-mediated mutagenesis as described [19]. Growth conditions and plant infections were performed as described [13]. Tumour tissue was harvested 10 days after plant inoculation for the isolation and subsequent germination of teliospores as described [13]. Strains were cultivated in YEPSl medium for the isolation of total DNA. If not further specified, all chemicals were of analytical grade and obtained from Sigma (Taufkirchen, Germany) or Roth (Karlsruhe, Germany).

### DNA procedures

Extraction of *U. maydis* total DNA and nucleic-acid procedures were performed as described [13,32]. Restriction enzymes were from NEB (New England Biolabs, Frankfurt a.M., Germany), oligonucleotides from MWG (Ebersberg, Germany). *U. maydis Δatg11* mutants were analysed either by diagnostic PCR or RFLP analysis as described [19]. For the verification of the *Δatg11* deletion the primer pair 5′-ACGAGAGCGGATCCGACAC-3′/5′-CGATCTTGTCGGAATGCAACG-3′ was used, which spans the *atg11* ORF from positions 3369 to 5468 bp. DNA fragments were labelled using the DIG-High Prime kit (Roche, Mannheim, Germany). Reagents for the detection of digoxigenin signals were from NEB.

### RFLP analysis

RFLP analysis of mitotypes using the digoxigenin-labelled 554-bp mtDNA-specific probe for detection was performed as described [16].

### PCR conditions

To discriminate between F, W and X1 mitotypes a diagnostic PCR was performed using primer combinations ‘a’ (5′-CACCATGGATACAACTTATGATTCTAC-3′/5′-TTTACGATAACGATTCATCGTCG-3′) and ‘b’ (5′-CACCATGAAAACACGACTATTTAATTTTAC-3′/5′-CAACGTGTAGTCTTTTTTAGTAG-3′), which are directed against the W type-specific LRII1 and the F type-specific LRI1 intron, respectively. Product lengths are 1018 bp (a) and 808 bp (b), respectively. PCR was performed using Taq (Roche) polymerase applying: 94°C, 3 min; 94°C, 25 s; 55°C, 20 s; 72°C, 1 min; 72°C, 10 min (30 repeats). PCR products were analysed by agarose (1%; w/v) gel electrophoresis (45 min, 100 V) in the presence of 0.01% (v/v) ethidium bromide.

### Quantitative real-time PCR (qPCR) analysis

qPCR analysis was performed using the Platinum SYBR qPCR Supermix-UDG kit (Life Technologies, Karlsruhe, Germany) in the presence of 10 nM fluorescein (Bio-Rad, Munich, Germany) and the intron-specific primer combinations 5′-TGATGATTGAACTACATGGTGTTGG-3′/5′-GAACTAAGTTTATGTCCACGAAGTG-3′ (141 bp product; F type intron LRI1) and 5′-GGAATTACCTGAATGATCTTCGTGA-3′/5′-CCGAAATTGAAATGATCCTTCTCCA-3′ (92 bp product; W type intron LRII1; [33]. Reactions contained 100 ng of total DNA as template and were run on an iCycler iQ (Bio-Rad). The used amount of DNA per reaction corresponds to ~5×10^6^ copies of nuclear DNA (20 Mb nuclear genome size; [34]). Based on relative intensities of mtDNA to nuclear DNA signals detected by Southern hybridization [16,17], the corresponding molecular ratio is estimated in the range of 10–100, equivalent to a maximum of 5×10^8^ mtDNA copies per reaction.

### Image analysis

Quantification of DNA bands (Tables 1 and 2) was performed using the ImageJ 1.48 software (http://imagej.nih.gov/ij/). The indication of faint F type signals in Figure 3 is based on recognition of peak profiles (see Figure 3B), with peak heights/widths >1.0. For defined plot parameters a square with lengths corresponding to the band width was laid over each potential band to be analysed.

### Statistical analysis

For non-parametric Mann–Whitney-*U*-test (two-tailed; α = 0,05) and boxplot analysis (95% confidence interval) the XLSTAT software was applied (www.xlstat.com). For paired student *t*-test analysis the SigmaPlot software was applied (http://www.sigmaplot.com).

## Additional files

Additional file 1: Table S1.
*U. maydis* strains used in this study. Additional file 2: Figure S1. Verification of the *Δatg11* deletion by RFLP analysis. A. Lanes 1, FB1Δatg11/pMB2-2; 2, GF5Δatg11/pKS1; 3, FB1/pMB2-2; 4, GF5/pKS1. B. Lanes 1, FB1/pMB2-2; 2, FB2/pMB2-2; 3, MF34/pKS2; 4, FB1Δatg11/pMB2-2; 5, FB2Δatg11/pMB2-2; 6, MF34Δatg11/pKS2. C. The schematic shows the restriction patterns of parental strains and generated null mutants (bottom). LF, left flank and RF, right flank (thick lines) bordering the region replaced by the *hyg* resistance cassette. Strains MF34, GF5 differ from strains FB1, FB2 by a XhoI restriction site polymorphism in the right border of *atg11* resulting in a larger fragment detected by RFLP analysis. The probe used for hybridization is depicted by the hatched box. Additional file 3: Figure S2. Analysis of pathogenicity of *U. maydis Δatg11* mutants. The strain combinations used for plant infections are: (1) MF34/pKS2 × FB2/pMB2-2, (2) MF34Δatg11/pKS2 × FB2Δatg11/pMB2-2, (3) FB1/pMB2-2 × GF5/pKS1, (4) FB1Δatg11/pMB2-2 × GF5Δatg11/pKS1. For (3), data were collected from two independent plant infections. Plants were inspected 10 days after inoculation. The severity of disease symptoms increases from top to bottom in the bar diagram. The x/y values on top of each column refer to the number of inspected plants and the disease indices calculated according to [35], respectively, with the categories as outlined in the figure. Additional file 4: Figure S3. RFLP analysis to detect mitotypes in wt and *Δatg11* spores (combination II). HindIII-digested DNA from individual spores from different tumour samples of the wt (FB1/pMB2-2 × GF5/pKS1) and the *Δatg11* mutant strain combination (FB1Δatg11/pMB2-2 × GF5Δatg11/pKS1). DNA from the parental strains K1 (FB1/pMB2-2), K3 (GF5/pKS1), K2 (FB1Δatg11/pMB2-2), and K4 (GF5Δatg11/pKS1) were included as controls. The arrows on the right refer to the marker bands W, X1 and F. Open circles refer to samples that are also contained in the PCR analysis (see Figure 3). Additional file 5: Figure S4. qPCR analysis to determine ratios of m2/m1 inheritance. A,B. qPCR analysis of samples (from a single tumour each) that either showed (arrowheads) or that did not show a faint F type band as detected by PCR analysis (see Figure 3, except for samples 19/1–19/4). Ratios of C_t_-values of W to F type are indicated. DNA from the parental strains K3 (GF5/pKS1) and K4 (GF5Δatg11/pKS1) were included as controls. The outer two lanes in (A) show ratios of C_t_-values of F to W type determined for the parental control strains K1 (FB1/pMB2-2) and K2 (FB1Δatg11/pMB2-2). Analyses shown in (A) and (B) were independently performed. Brackets are labelled (ns, non-significant; *p-value <0.05; paired student *t*-test) in case the bar marked with an arrowhead is smaller than the reference bar within a bracket. For the data pair in [1/1,1/2], 1/1 was taken as reference because of its hardly detectable F type signal (see Figure 3A).

## Abbreviations

UPI: Uniparental inheritance
MT: Mitochondrial
qPCR: Quantitative real-time PCR analysis
WT: Wild-type

## References

[1] Birky CW (2001). The inheritance of genes in mitochondria and chloroplasts: laws, mechanisms, and models. Annu Rev Genet.

[2] Birky CW (2008). Uniparental inheritance of organelle genes. Curr Biol.

[3] Xu J (2005). The inheritance of organelle genes and genomes: patterns and mechanisms. Genome.

[4] Basse CW (2010). Mitochondrial inheritance in fungi. Curr Opin Microbiol.

[5] Gyawali R, Lin X (2013). Prezygotic and postzygotic control of uniparental mitochondrial DNA inheritance in *Cryptococcus neoformans*. MBio.

[6] Bendich AJ (2013). DNA abandonment and the mechanisms of uniparental inheritance of mitochondria and chloroplasts. Chromosome Res.

[7] Al Rawi S, Louvet-Vallée S, Djeddi A, Sachse M, Culetto E, Hajjar C (2011). Postfertilization autophagy of sperm organelles prevents paternal mitochondrial DNA transmission. Science.

[8] Sato M, Sato K (2011). Degradation of paternal mitochondria by fertilization-triggered autophagy in *C. elegans* embryos. Science.

[9] Levine B, Elazar Z (2011). Development. Inheriting maternal mtDNA. Science.

[10] Sato M, Sato K (2012). Maternal inheritance of mitochondrial DNA: degradation of paternal mitochondria by allogeneic organelle autophagy, allophagy. Autophagy.

[11] Nakatogawa H, Suzuki K, Kamada Y, Ohsumi Y (2009). Dynamics and diversity in autophagy mechanisms: lessons from yeast. Nat Rev Mol Cell Biol.

[12] Youle RJ, Narendra DP (2011). Mechanisms of mitophagy. Nat Rev Mol Cell Biol.

[13] Fedler M, Luh KS, Stelter K, Nieto-Jacobo F, Basse CW (2009). The *a2* mating-type locus genes *lga2* and *rga2* direct uniparental mitochondrial DNA (mtDNA) inheritance and constrain mtDNA recombination during sexual development of *Ustilago maydis*. Genetics.

[14] Kahmann R, Steinberg G, Basse C, Feldbrügge M, Kämper J, Kronstad JW (2000). *Ustilago maydis*, the causative agent of corn smut disease. Fungal Pathology.

[15] Brefort T, Doehlemann G, Mendoza-Mendoza A, Reissmann S, Djamei A, Kahmann R (2009). *Ustilago maydis* as a Pathogen. Annu Rev Phytopathol.

[16] Bortfeld M, Auffarth K, Kahmann R, Basse CW (2004). The *Ustilago maydis a2* mating-type locus genes *lga2* and *rga2* compromise pathogenicity in the absence of the mitochondrial p32 family protein Mrb1. Plant Cell.

[17] Mahlert M, Vogler C, Stelter K, Hause G, Basse CW (2009). The *a2* mating-type-locus gene *lga2* of *Ustilago maydis* interferes with mitochondrial dynamics and fusion, partially in dependence on a Dnm1-like fission component. J Cell Sci.

[18] Urban M, Kahmann R, Bölker M (1996). Identification of the pheromone response element in *Ustilago maydis*. Mol Gen Genet.

[19] Nieto-Jacobo F, Pasch D, Basse CW (2012). The mitochondrial Dnm1-like fission component is required for *lga2*-induced mitophagy but dispensable for starvation-induced mitophagy in *Ustilago maydis*. Eukaryot Cell.

[20] Kanki T, Klionsky DJ (2008). Mitophagy in yeast occurs through a selective mechanism. J Biol Chem.

[21] Kanki T, Klionsky DJ (2010). The molecular mechanism of mitochondria autophagy in yeast. Mol Microbiol.

[22] Kämper J (2004). A PCR-based system for highly efficient generation of gene replacement mutants in *Ustilago maydis*. Mol Genet Genomics.

[23] Sutovsky P, Moreno RD, Ramalho-Santos J, Dominko T, Simerly C, Schatten G (1999). Ubiquitin tag for sperm mitochondria. Nature.

[24] Luo SM, Sun QY (2013). Autophagy is not involved in the degradation of sperm mitochondria after fertilization in mice. Autophagy.

[25] Luo SM, Ge ZJ, Wang ZW, Jiang ZZ, Wang ZB, Ouyang YC (2013). Unique insights into maternal mitochondrial inheritance in mice. Proc Natl Acad Sci U S A.

[26] Nadal M, Gold SE (2010). The autophagy genes *atg8* and *atg1* affect morphogenesis and pathogenicity in *Ustilago maydis*. Mol Plant Pathol.

[27] Westermann B (2010). Mitochondrial fusion and fission in cell life and death. Nat Rev Mol Cell Biol.

[28] Mao K, Wang K, Liu X, Klionsky DJ (2013). The scaffold protein Atg11 recruits fission machinery to drive selective mitochondria degradation by autophagy. Dev Cell.

[29] Liu L, Sakakibara K, Chen Q, Okamoto K (2014). Receptor-mediated mitophagy in yeast and mammalian systems. Cell Res.

[30] Shakya VP, Idnurm A (2014). Sex determination directs uniparental mitochondrial inheritance in phycomyces. Eukaryot Cell.

[31] Wilch G, Ward S, Castle A (1992). Transmission of mitochondrial DNA in *Ustilago violacea*. Curr Genet.

[32] Basse CW, Kolb S, Kahmann R (2002). A maize-specifically expressed gene cluster in *Ustilago maydis*. Mol Microbiol.

[33] Pfeifer A, Martin B, Kämper J, Basse CW (2012). The mitochondrial LSU rRNA group II intron of *Ustilago maydis* encodes an active homing endonuclease likely involved in intron mobility. PLoS One.

[34] Kämper J, Kahmann R, Bölker M, Ma LJ, Brefort T, Saville BJ (2006). Insights from the genome of the biotrophic fungal plant pathogen *Ustilago maydis*. Nature.

[35] Kronstad JW, Leong SA (1989). Isolation of two alleles of the *b* locus of *Ustilago maydis*. Proc Natl Acad Sci U S A.

