# Supplementary material for: Uniparental mitochondrial DNA inheritance is not affected in Ustilago maydis Δatg11 mutants blocked in mitophagy
Source: BMC Microbiol. 2015 Feb 6;15(1):23. doi: 10.1186/s12866-015-0358-z (PMC4326477; doi:10.1186/s12866-015-0358-z)
Supplement: Additional file 1: Table S1. — U. maydis strains used in this study. [file 12866_2015_358_MOESM1_ESM.pdf]

Additional file 1

Table S1. ***U. maydis*** **strains used in this study**

Strain	Genotype	Mitotype	Reference
BUB7	*a1 b3*	X1	[32]
FB2/pMB2-2	*a2 b2 Potef-mtGFP-cbx*^{R,1}	F	[16]
FB2Δatg11/pMB2-2	*a2 b2 atg11::hyg*^{R} *Potef-mtGFP-cbx*^{R}	F	[19]
MF34/pKS2	*a1 b14 Pcrg1-mtRFP- cbx*^{R,2}	W	[13]
MF34Δatg11/pKS2	*a1 b14 atg11::hyg*^{R} *Pcrg1-mtRFP-cbx*^{R}	W	This study
FB1/pMB2-2	*a1 b1 Potef-mtGFP-cbx*^{R}	F	[16]
FB1Δatg11/pMB2-2	*a1 b1 atg11::hyg*^{R} *Potef-mtGFP-cbx*^{R}	F	[19]
GF5/pKS1	*a2 b13 Pcrg1-mtGFP-cbx*^{R}	W	[13]
GF5Δatg11/pKS1	*a2 b13 atg11::hyg*^{R} *Pcrg1-mtGFP-cbx*^{R}	W	This study

cbx: carboxin resistance cassette; hyg: hygromycin resistance cassette.

[1]*Potef*: expression under the constitutive *otef* promoter.

[2]*Pcrg1*: expression under the inducible *crg1* promoter.
