# Supplementary figures and images for: Uniparental mitochondrial DNA inheritance is not affected in Ustilago maydis Δatg11 mutants blocked in mitophagy
Source: BMC Microbiol. 2015 Feb 6;15(1):23. doi: 10.1186/s12866-015-0358-z (PMC4326477; doi:10.1186/s12866-015-0358-z)

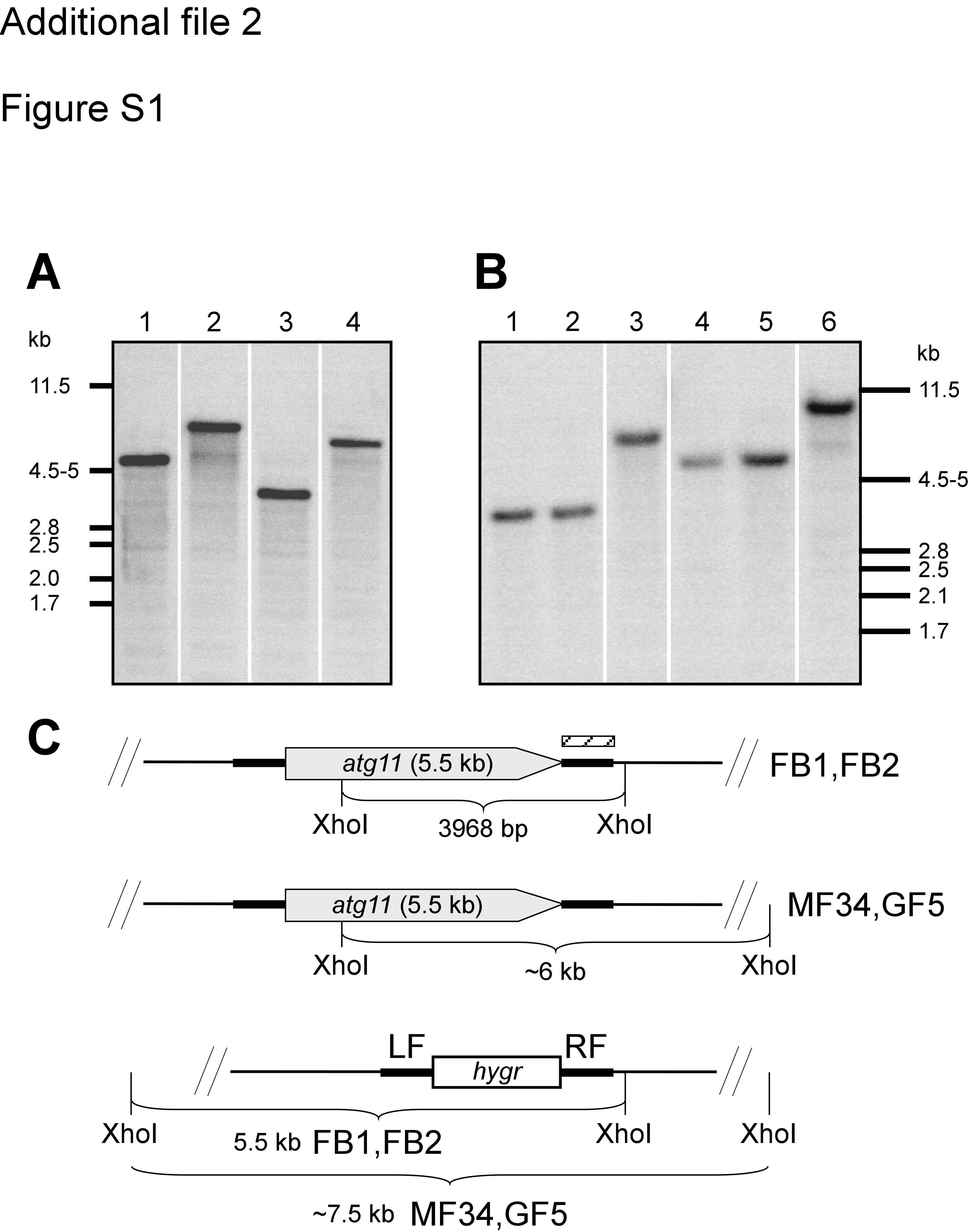

Supplement: Additional file 2: Figure S1. — Verification of the Δatg11 deletion by RFLP analysis. A. Lanes 1, FB1Δatg11/pMB2-2; 2, GF5Δatg11/pKS1; 3, FB1/pMB2-2; 4, GF5/pKS1. B. Lanes 1, FB1/pMB2-2; 2, FB2/pMB2-2; 3, MF34/pKS2; 4, FB1Δatg11/pMB2-2; 5, FB2Δatg11/pMB2-2; 6, MF34Δatg11/pKS2. C. The schematic shows the restriction patterns of parental strains and generated null mutants (bottom). LF, left flank and RF, right flank (thick lines) bordering the region replaced by the hyg resistance cassette. Strains MF34, GF5 differ from strains FB1, FB2 by a XhoI restriction site polymorphism in the right border of atg11 resulting in a larger fragment detected by RFLP analysis. The probe used for hybridization is depicted by the hatched box. [file 12866_2015_358_MOESM2_ESM.tiff]

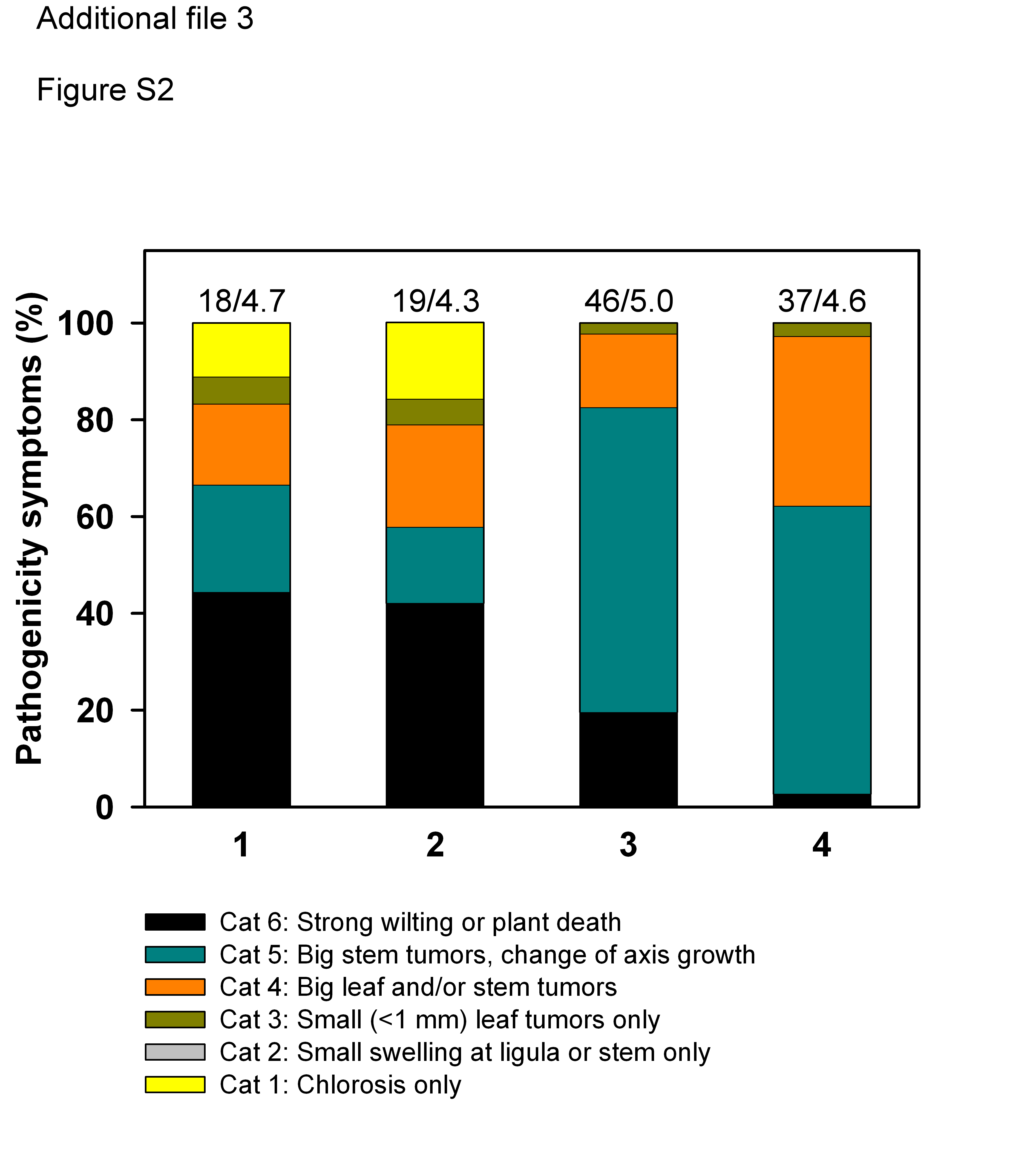

Supplement: Additional file 3: Figure S2. — Analysis of pathogenicity of U. maydis Δatg11 mutants. The strain combinations used for plant infections are: (1) MF34/pKS2 × FB2/pMB2-2, (2) MF34Δatg11/pKS2 × FB2Δatg11/pMB2-2, (3) FB1/pMB2-2 × GF5/pKS1, (4) FB1Δatg11/pMB2-2 × GF5Δatg11/pKS1. For (3), data were collected from two independent plant infections. Plants were inspected 10 days after inoculation. The severity of disease symptoms increases from top to bottom in the bar diagram. The x/y values on top of each column refer to the number of inspected plants and the disease indices calculated according to [35], respectively, with the categories as outlined in the figure. [file 12866_2015_358_MOESM3_ESM.tiff]

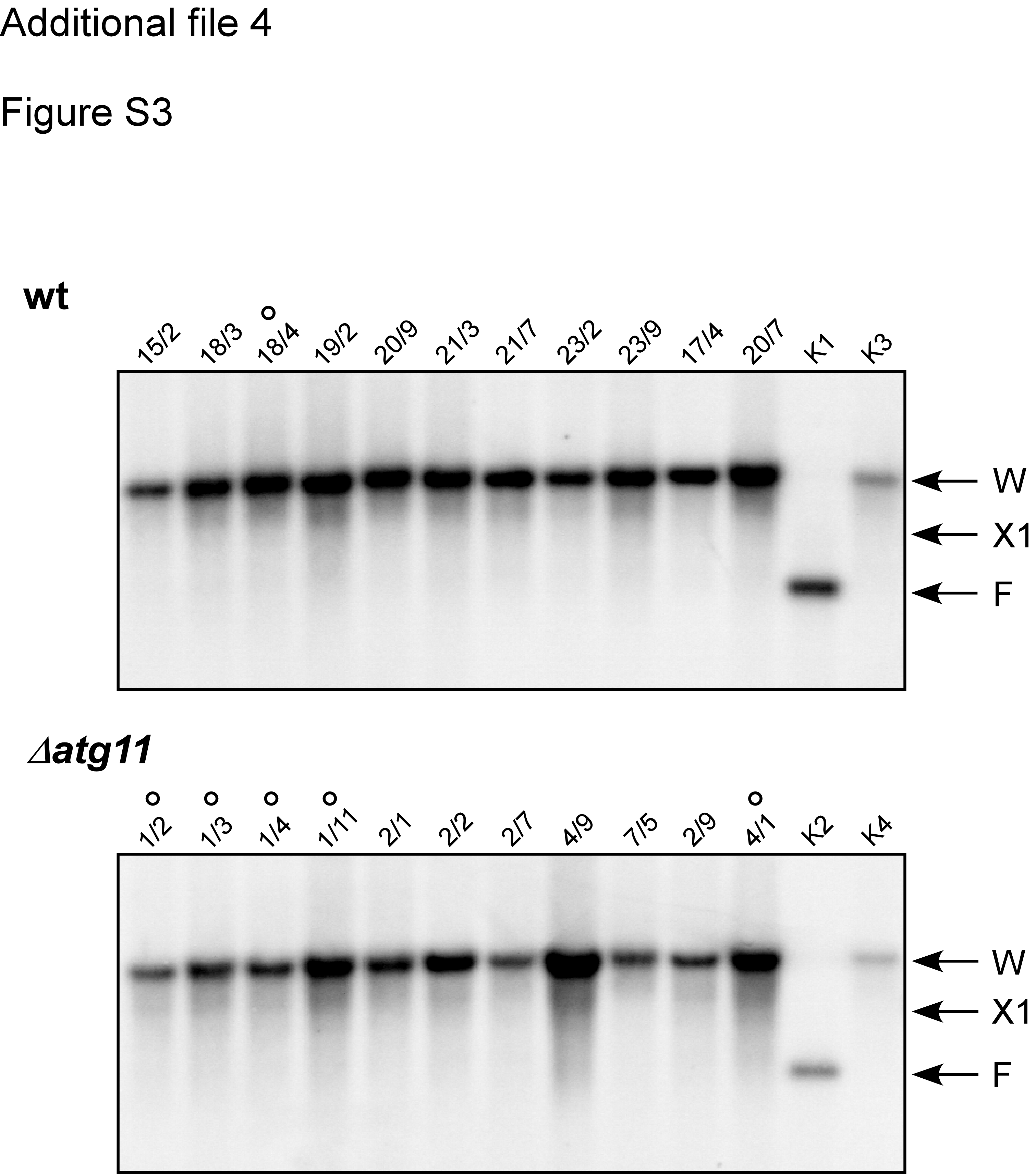

Supplement: Additional file 4: Figure S3. — RFLP analysis to detect mitotypes in wt and Δatg11 spores (combination II). HindIII-digested DNA from individual spores from different tumour samples of the wt (FB1/pMB2-2 × GF5/pKS1) and the Δatg11 mutant strain combination (FB1Δatg11/pMB2-2 × GF5Δatg11/pKS1). DNA from the parental strains K1 (FB1/pMB2-2), K3 (GF5/pKS1), K2 (FB1Δatg11/pMB2-2), and K4 (GF5Δatg11/pKS1) were included as controls. The arrows on the right refer to the marker bands W, X1 and F. Open circles refer to samples that are also contained in the PCR analysis (see Figure 3). [file 12866_2015_358_MOESM4_ESM.tiff]

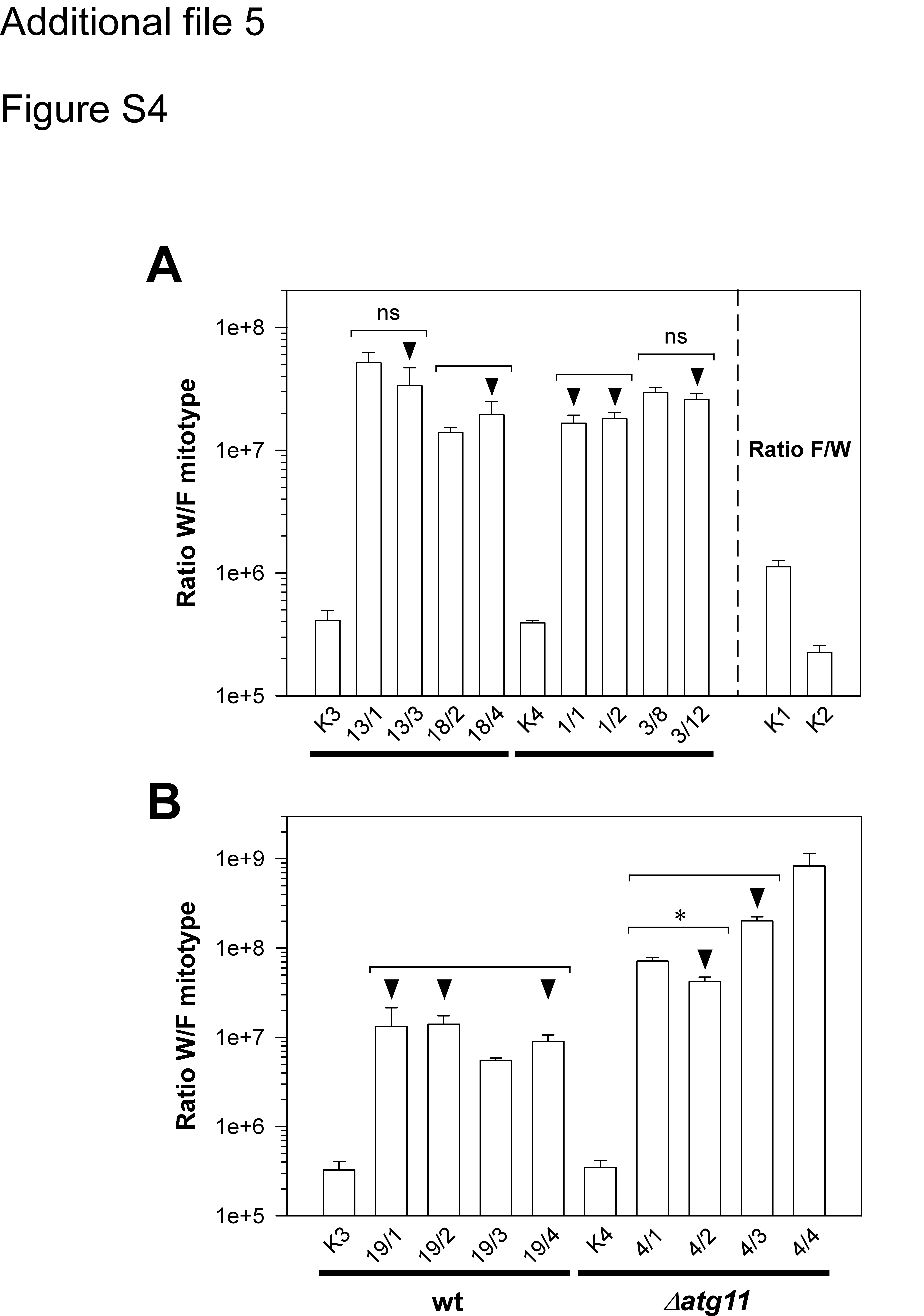

Supplement: Additional file 5: Figure S4. — qPCR analysis to determine ratios of m2/m1 inheritance. A,B. qPCR analysis of samples (from a single tumour each) that either showed (arrowheads) or that did not show a faint F type band as detected by PCR analysis (see Figure 3, except for samples 19/1–19/4). Ratios of Ct-values of W to F type are indicated. DNA from the parental strains K3 (GF5/pKS1) and K4 (GF5Δatg11/pKS1) were included as controls. The outer two lanes in (A) show ratios of Ct-values of F to W type determined for the parental control strains K1 (FB1/pMB2-2) and K2 (FB1Δatg11/pMB2-2). Analyses shown in (A) and (B) were independently performed. Brackets are labelled (ns, non-significant; *p-value <0.05; paired student t-test) in case the bar marked with an arrowhead is smaller than the reference bar within a bracket. For the data pair in [1/1,1/2], 1/1 was taken as reference because of its hardly detectable F type signal (see Figure 3A). [file 12866_2015_358_MOESM5_ESM.tiff]
